# Concurrent anti-neutrophil cytoplasmic antibody-associated glomerulonephritis and IgG4-associated tubulointerstitial nephritis with C3 glomerulonephritis: A case report

**DOI:** 10.1097/MD.0000000000018857

**Published:** 2020-01-31

**Authors:** Jianan Feng, Jinyu Yu, Xueyao Wang, Yue Wang, Yang Liu, Zhonggao Xu, Weixia Sun

**Affiliations:** a Department of Nephrology; b Department of Ultrasound, The First Hospital of Jilin University, Changchun, Jilin, China.

**Keywords:** ANCA-associated glomerulonephritis, C3 glomerulonephritis, IgG4-associated tubulointerstitial nephritis

## Abstract

**Rationale:**

IgG4-related disease (IgG4-RD) is a slowly progressing inflammatory disease that can involve multiple organ systems. There is considerable overlap between IgG4-RDs and anti-neutrophil cytoplasmic antibody (ANCA)-associated vasculitis (AAV). Herein, we present an unusual case of IgG4-associated tubulointerstitial nephritis (IgG4-TIN) and ANCA-associated glomerulonephritis (ANCA-GN) co-occurring with C3 glomerulonephritis (C3GN).

**Patient concerns:**

A 72-year-old male was admitted to hospital because of fever and fatigue. He was diagnosed with elevated serum creatinine and IgG4 levels, and was positive for ANCA.

**Diagnosis:**

Initially, the pathology supported a diagnosis of IgG4-TIN and ANCA-GN; however, further examination revealed he also had C3GN.

**Interventions:**

The patient was treated with methylprednisolone and cyclophosphamide and received regular follow-up care.

**Outcomes:**

After treatment, the patient no longer exhibited fever or fatigue and had no complications. The seven-month follow-up showed downward trends in IgG4 and MPO-ANCA levels and stable 24-hour urine protein, serum creatinine levels.

**Lessons:**

Anti-neutrophil cytoplasmic antibody-associated glomerulonephritis and IgG4-associated tubulointerstitial nephritis with C3glomerulonephritis rarely occur simultaneously. Laboratory analysis and pathology are both needed to ensure diagnostic accuracy. However, in this case, the three diseases overlapped to such a large extent that achieving a definitive diagnosis was particularly challenging. Timely and accurate diagnosis is crucial for selecting the best treatment course and optimizing patient outcome.

## 1 Introduction

IgG4-related disease (IgG4-RD) is a fibro-inflammatory condition that can affect every organ system in the body.^[1]^ The diagnosis of IgG4-RD is challenging: in fact, a range of organs can be affected and the clinical, serological, and histological findings can be heterogeneous.^[2]^ There is substantial overlap in the types of organs involved and the histopathology of IgG4-RD and anti-neutrophil cytoplasmic antibody (ANCA)-associated vasculitis (AAV).^[3]^ C3 glomerulonephritis (C3GN) was first characterized by Verroust in 1974 as a type of glomerulopathy, named by Fakhoufi et al, that is characterized by immunoreactive C3 complement protein deposited along the capillary loop or mesangial area of glomeruli, without immunoglobulin deposition.^[4]^ There are no reports of co-occurrence of these three kidney diseases in a single patient. Here, we describe a case of ANCA-GN and IgG4-TIN with C3GN simultaneously affecting 1 patient. We analyzed the clinical characteristics, pathology, common pathophysiologies, and histopathological characteristics of ANCA-GN, IgG4-TIN, and C3GN to provide clinical insight and facilitate more accurate clinical diagnoses.

## 2 Case description

A 72-year-old male was admitted to our hospital after experiencing 2 months of intermittent fever, fatigue, and full-body discomfort. He was treated with antibiotics at a local hospital, but there was no improvement in his symptoms. The fever was apparently unprovoked. He had no known personal or family history of renal dysfunction. Physical examination indicated his blood pressure was 137/74 mmHg, heart rate was 100 beats/min, and cardiopulmonary and neurological functions were normal, with no costovertebral knocking tenderness. A urinalysis performed upon admission showed 3+ occult blood and his 24-hour urine protein level was 1.24 g. Serum biochemistry revealed the following: creatinine, 233.9 μmol/L; MPO-ANCA, 203.45 AU/mL, p-ANCA, 1:32, IgG, 22.90 g/L; IgG4, 7.230 g/L; complement C3, 0.82 g/L, erythrocyte sedimentation rate (ESR), 120 mm/h; and C-reactive protein (CRP), 141 mg/L. Routine blood analysis revealed the following: white blood cells, 11.45 x 10^9^/L; hemoglobin, 72 g/L, and platelets, 400 x 10^9^/L. Abdominal CT results were normal. A bone marrow smear showed signs of hyperplastic anemia. Granulocyte-poisoning particles in peripheral blood, vacuole degeneration, and, occasionally, late granulocytes were also present.

Among sixteen glomeruli examined in a renal biopsy, one had glomerular sclerosis, and eight had cellular crescent formations, we observed 2 cellular fibrinous crescents, and one renal bulb with segmental celluloid necrosis. Furthermore, there was evidence of local mild edema and diffuse inflammatory cell infiltration into the renal stroma, predominantly lymphocytes and plasma cells, macrophages, a small number of neutrophils and eosinophils, in addition to stromal foci with mild fibrosis. Immunofluorescent staining of three glomeruli indicated that these were highly C3-immunoreactive yet negative for IgA, IgM, IgG, C4, and complement F. Immunohistochemical analysis demonstrated that most of the IgG-positive infiltrating inflammatory cells were IgG4 immunoreactive, accounting for over 40% of the IgG-positive cells.

**Figure 1:**
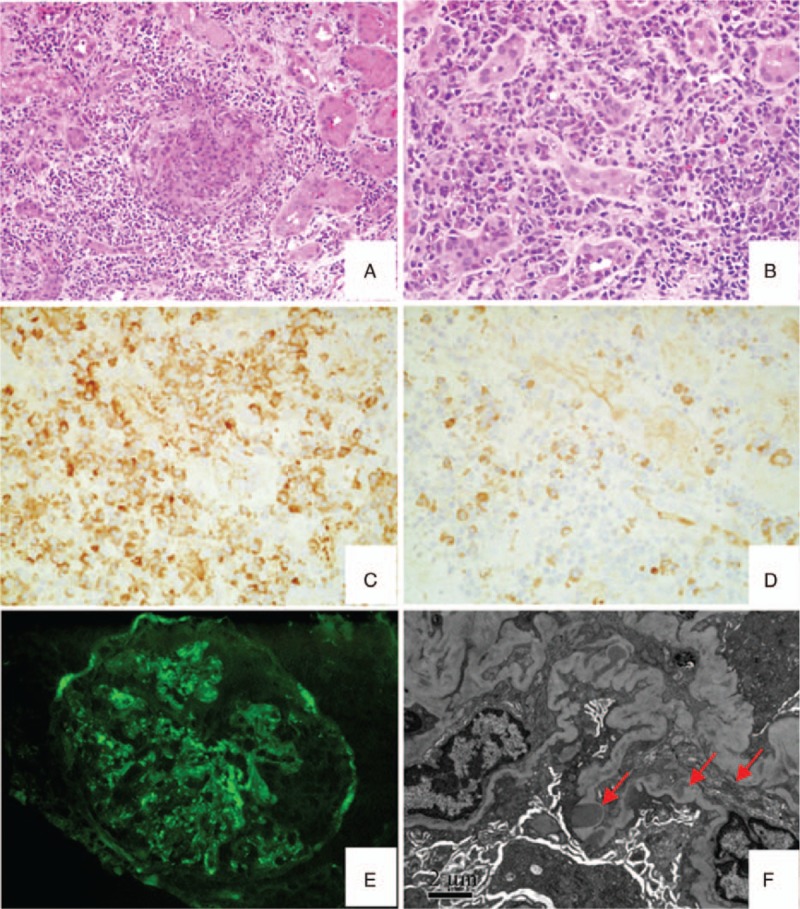
Pathological findings in the renal biopsy specimen. (A) Necrotizing crescentic glomerulonephritis (HE 200×). (B) Plasma cell infiltrates in the renal interstitium (HE 400×). (C, D) IgG-positive and IgG4-positive plasma cell infiltrates in the renal intersitium (immunohistochemical staining, 400X). (E) Glomerular immunofluorescence showing diffuse granular mesangial deposition of C3 (400X). (F) Electron microscopy image demonstrating subepithelial intramembranous and mesangial deposits (red arrow).

Our patient was admitted due to intermittent fever and fatigue. Upon admission, further testing revealed albuminuria, hematuria, elevated serum creatinine levels, serum IgG4 antibody levels three-fold higher than typical healthy values, and MPO-ANCA-positive serum. These serological indicators are characteristic of IgG4-TIN and ANCA-GN. The pathological results from a renal puncture indicated that 67% of the glomeruli were marked by crescent-shaped scars with segmental cellulose-like necrosis, with no obvious immune deposits, which is in line with the pathological characteristics of ANCA-GN (Fig. 1A). The renal stroma was slightly fibrotic with IgG4-positive plasma cell (representing more than 40% of IgG-positive cells) and mild eosinophil infiltration, which is in accordance with the diagnostic criteria for IgG4-RD (Fig. 1B–D). Additionally, immunofluorescence revealed diffuse granular mesangial deposition of C3 (Fig. 1E) and electron microscopy showed electron-dense sediment deposition in the subepithelial intramembranous and mesangial regions of the kidney (Fig. 1F). Therefore, the patient was finally diagnosed with ANCA-GN and IgG4-TIN with C3GN.

**Figure 2:**
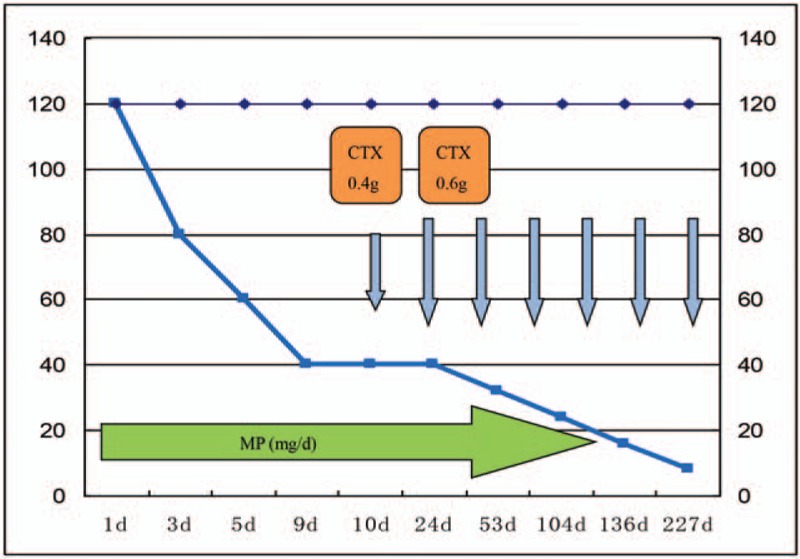
Therapeutic course of methylprednisolone (MP) and cyclophosphamide (CTX). The initial dose of methylprednisolone (delivered orally) was 120 mg/d, which was reduced to 80 mg/d, 60 mg/d, and then 40 mg/d, before finally being finally reduced gradually to maintenance levels. On the tenth day of MP therapy, cyclophosphamide was added. The initial dose of cyclophosphamide was 0.4 g. Fifteen days later,the dose was increased to 0.6 g per month.

We prescribed methylprednisolone and cyclophosphamide (Fig. 2). The patient had a history of tuberculosis and, therefore, rifampicin and isoniazid were also prescribed to prevent its reactivation and dissemination. Additionally, the patient was treated with Adalat, a calcium antagonist used for the treatment of hypertension, to manage his blood pressure and Recombinant Human Erythropoietin to improve anemia.

**Figure 3:**
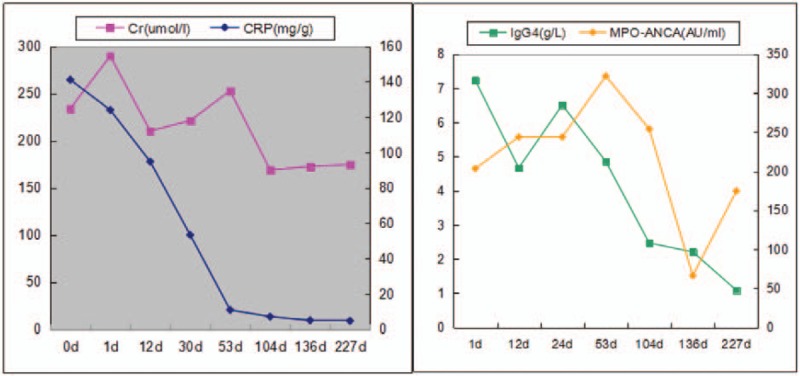
Clinical course after admission. C-reactive protein (CRP); creatinine (Cr).

He continued with these treatments and received regular follow-up care at our hospital. The monthly follow-up showed downward trends in MPO-ANCA, 24-hour urine protein, IgG4, CRP, and ESR, but creatinine remained around 170 μmol/L (Fig. 3). Due to eye discomfort, the patient stopped taking methylprednisolone 6 months after initiating treatment. In the seven month follow-up period, no recurrence of the disease was observed.

## 3 Discussion

Given the presence of autoantibodies, IgG4-RD is categorized as an autoimmune disease; however, there is no evidence that these autoantibodies contribute directly to the pathogenesis or progression of the disease. IgG4-RD lesions are infiltrated by T helper cells, which likely cause progressive fibrosis and organ damage. The pathogenesis of the disease is not clear.^[5]^ The histological hallmarks characteristic of IgG4-RD include storiform fibrosis, obliterative phlebitis, tissue eosinophilia, and lymphoplasmacytic infiltration in which the ratio of IgG4- to IgG-positive plasma cells usually exceeds 0.40; interestingly, these features also affect seemingly unrelated organs.^[6]^ To date, diagnostic criteria for IgG4-RD have been proposed by two groups.^[7,8]^ IgG4-RD generally responds to glucocorticoids during its inflammatory stage, but recurrent or refractory cases are common. The extent of the fibrosis is a useful predictor of responsiveness to immunosuppressive therapies.^[9,10]^

‘IgG4-related tubulointerstitial nephritis (IgG4-TIN)’ was first proposed by Saeki T.^[11]^ Patients with IgG4-TIN generally present with proteinuria, hematuria, decreased kidney function, hypocomplementemia, and radiologic abnormalities. IgG4-TIN has the same histopathology as in other organs, however, it can be distinguished from many other forms of organ involvement by the presence of extremely low concentrations of complement protein. One plausible explanation is that hypocomplementaemia in IgG4-RD results from the formation of immune complexes that contain IgG1 or IgG3, which bind complement more effectively,^[10]^ which supports the notion that IgG4 does not directly contribute to the pathogenesis of IgG4-TIN.

AAV is a heterogeneous group of diseases that show necrotizing inflammation of small vessels with a wide range of clinical presentations and includes: granulomatosis with polyangiitis (GPA), eosinophilic GPA (EGPA), and microscopic polyangiitis (MPA).^[12]^ Kidney involvement seems to be more frequent in MPA than GPA but some groups have not been able to confirm this result. By contrast, ear nose and throat involvement is more frequent in GPA than MPA.^[13]^ In ANCA-GN, immunoglobulin deposition in glomeruli is considered to be infrequent and minimal and, therefore, it is classified as pauci-immune glomerulonephritis.^[14]^ However, studies have shown granular IgG and/or IgM deposition in ANCA-GN.^[15]^ MPO-ANCA-GN is characterized by pauci-immune necrotizing glomerulonephritis (NGN) with crescent formation.^[16]^ MPO-ANCAs bind to MPO molecules on the surface of primed neutrophils/monocytes and induce the release of reactive oxygen species and proteases near the endothelium, thereby causing glomerular and systemic capillary inflammation.^[17]^ Recent studies have also implicated other factors, including immunoglobulins, that are associated with glomeruli.^[18]^ AAV can eventually lead to acute renal failure, life-threatening pulmonary hemorrhage, and gastrointestinal dysfunction and, as such, are potentially fatal.^[19]^

The similarities between the organs affected, biochemistry, and pathogenesis of IgG4-RD and AAV warrants elaboration.

1) IgG4-RD in patients who are ANCA-positive is limited to the head and neck and does not involve other commonly affected organs, such as the lungs, pancreas, biliary tree, and retroperitoneum. By contrast, AAV in patients with established IgG4-RD presents with systemic manifestations beyond the ear/nose/throat region. One case study reported a patient with histological features of the left lacrimal gland that were diagnostic for IgG4-RD while the patient's lung biopsy showed typical features of GPA.^[3]^
2) Although serum IgG4 is regarded as a sensitive marker of IgG4-RD, it lacks diagnostic specificity.^[20]^ IgG4 infiltration is not unique to IgG4-RD. Tao Su et al reported a case of typical IgG4-TIN concurrent with IgG4 MPO-ANCA-positive necrotizing crescentic GN.^[21]^ Raissian et al reported that moderate to marked IgG4-positive plasma cell infiltration is observed in 31.7% of patients with pauci-immune vasculitis,^[9]^ especially in MPO-ANCA-associated EGPA or Churg–Strauss syndrome.^[7]^ Moreover, ANCA is sometimes absent in AAV cases. Toru Sakairi et al reported an ANCA-negative case with histological and clinical findings similar to those seen in IgG4-TIN; however, the presence of cellular crescent formation in a few glomeruli and fibrinoid necrosis in an interlobular artery led to a diagnosis of small-vessel vasculitis, which is considered to be a renal manifestation of AAV.^[22]^ Serological indicators are not sufficient to define a disease; rather, to achieve a definitive diagnosis, these must be combined with pathological features.
3) IgG is a potent neutrophil stimulus, particularly when presented as ANCAs in AAV. Normal IgG binds flowing neutrophils efficiently with an affinity in the order of IgG3 > IgG1 > IgG2 > IgG4. Patients with IgG4-RD exhibit diffuse infiltration of IgG4-producing plasma cells into the interstitial renal space. One study reported that IgG1 and IgG4 were dominant in both MPO-ANCA-positive IgG preparations and PR3-ANCA.^[23]^ Whether IgG4 can induce an autoantigen-mediated secondary inflammatory response requires further study.

C3GN is a rare disorder characterized by excess alternative complement pathway activation.^[24]^ C3GN affects all ages, and does not have a sex predilection. Most patients present with hypertension, proteinuria, and hematuria. In both the short- and long-term, renal function remains stable in the majority of patients.^[25]^ Alternative pathway abnormalities are heterogeneous and can have both acquired and genetic origins. The most common acquired abnormality is the presence of C3 nephritic factors (C3Nef), while the most common genetic contribution arises from the H402 and V62 alleles of factor H. In addition, other risk factors include factor H autoantibodies and mutations in CFH, CFI, and CFHR genes. These factors result in the dysregulation of the alternative pathway of complement activation, causing aberrant complement protein deposition on glomeruli and, consequently, glomerular injury.^[25]^ Light microscopy-based studies of C3 glomerulonephritis have demonstrated its proliferative pattern. Immunohistochemistry studies have revealed a dominant C3 staining pattern with minimal or no Ig immunoreactivity. Electron microscopy studies have shown that C3 deposits are located in the mesangium and along the capillary wall.^[24,26]^ Although immunosuppressive agents and terminal complement pathway blockers are beneficial for some patients, there is no treatment for this disease. Limited data from patients with renal transplants indicate that the recurrence rate of C3GN is high.^[27,28]^

Studies have shown that C3GN and AAV overlap in their pathogenesis. Hillhorst et al retrospectively analyzed 187 renal biopsies from patients with ANCA-positive glomerulonephritis. Among these, 78 stained positive for C3 complement protein using immunohistochemistry. They found that complement activation in AAV occurs predominantly via the alternative pathway.^[29]^ In C3GN, C3Nefs can stabilize C3 inverters by prolonging their half-life, increasing C3 fragmentation, and thereby decreasing serum complement C3 levels; moreover, abnormal complement regulatory proteins can also lead to excessive activation of complement alternative pathways.^[30]^ Aadel Chaudhuri et al reported a case of C3GN co-occurring with GPA, but this diagnosis remains unclear as the incidence of GPA is high and other reports have described cases of GPA with glomerular immune deposits. Therefore, it is difficult to determine whether this patient truly harbored both diseases or a variant of only one of the diseases. This case nicely demonstrates the pathogenic similarities between AAV and C3GN; however, the specificities of the underlying mechanisms require further investigation.^[31,32]^

To our knowledge, this the first reported case of a patient with concurrent IgG4-TIN, ANCA-GN, and C3GN. This rare diagnosis was substantiated by several factors. The patient's IgG4 level was above normal and he was positive for MPO-ANCA. Pathology findings included IgG4-positive plasma cell infiltration, crescent-shaped glomeruli, deposition of immunoreactive C3, and the presence of electron-dense sediment in the superior subcutaneous region, basement membrane, and mesangial area. Taken together, these clinical features led us to diagnose concurrent IgG4-TIN and MPO-ANCA-associated necrotizing crescentic GN with C3GN. The patient responded well to methylprednisolone and cyclophosphamide treatment with marked improvement in renal function.

## 4 Conclusions

In conclusion, here we describe a rare case of concurrent IgG4-TIN and MPO-ANCA-associated necrotizing crescentic GN with C3GN. This case exemplifies the difficulty of differentially diagnosing three separate diseases and also provides insight into the relationship between IgG4-RD and AAV and how to accurately identify them.^[6,16,19,29,31,32]^

## Author contributions

**Conceptualization:** Jianan Feng, Weixia Sun

**Data curation:** Jinyu Yu, Xueyao Wang

**Funding acquisition:** Weixia Sun

**Investigation:** Yue Wang, Yang Liu

**Project administration:** Zhonggao Xu

**Resources:** Weixia Sun

**Software:** Jinyu Yu, Xueyao Wang

**Validation:** Weixia Sun

**Visualization:** Jianan Feng

**Writing – original draft:** Jianan Feng

**Writing – review & editing:** Weixia Sun
